# Immunotherapy: should we worry about immunosenescence?

**DOI:** 10.70401/acrt.2024.104

**Published:** 2024-04-22

**Authors:** Asli Özkan, Nienke A. de Glas, Johanneke E.A. Portielje

**Affiliations:** 1 Department of Medical Oncology, Department of Medical Oncology, Leiden University Medical Center, Leiden 2333 ZA, The Netherlands; 2 Department of Medical Oncology, Helse Førde, Svanehaugvegen 2, Førde 6812, Norway

**Keywords:** Immunosenescence, immunotherapy, checkpoint inhibitors, oncology, frailty

## Abstract

The global aging population is expected to experience a nofigure increase in cancer incidence, particularly among individuals aged 70 and older. At the same time, the extensive use of immune checkpoint inhibitors (ICIs) in cancer treatment raises questions about the influence of immunosenescence, the age-related decline in immune function, on treatment efficacy in older patients. Despite promising outcomes, resistance to immunotherapies and the occurrence of severe immune-related adverse events (irAEs) remain challenges. Limited research has explored the correlation between immunosenescence markers in peripheral blood and the tumour microenvironment (TME), frailty, and ICI response, and irAEs in older patients. This commentary explores the interrelationship between immunosenescence and immunotherapy in older and frail patients with cancer undergoing ICI therapy. Understanding the impact of immunosenescence on treatment response and irAEs, and identifying reliable biomarkers, is crucial for future research in geriatric oncology, as this will possibly facilitate patient stratification and personalized treatment approaches, ultimately improving patient outcomes while minimizing irAE-related risks.

## 1 Introduction

In the coming decades, the incidence of cancer among the older population will increase significantly, especially among those aged over 70. This upward trend is a direct result of significant medical advances made over the last century, especially in high-income countries, with considerably increased life expectancy. Projections indicate that the global population of individuals aged over 80 years is poised to triple, surging from 143 million worldwide in 2019 to an estimated 426 million by 2050^[1]^. This demographic shift indicates an impending rise in the prevalence of cancer, particularly affecting the older population.

The introduction of immune checkpoint inhibitors (ICIs) in 2011 revolutionized cancer treatment, marking a significant milestone in the realm of oncology. ICIs operate by targeting immune checkpoints on immune cells, thereby regulating the immune system’s response^[2]^. Checkpoint proteins, such as Programmed Cell Death protein 1 (PD-1) on T cells and its ligand (PD-L1) on tumour cells, regulate immune responses to prevent overactivity. The interaction between PD-1 and PD-L1 inhibits T cells from attacking tumour cells. By blocking this interaction with immune checkpoint inhibitors (anti-PD-1 or anti-PD-L1), T cells are able to effectively target and kill tumour cells^[3]^. The two major types of immune checkpoint proteins targeted by ICIs are Cytotoxic T-Lymphocyte-Associated Protein 4 (CTLA-4, e.g., ipilimumab) and PD-1/PD-L1 (e.g., nivolumab and pembrolizumab)^[2]^. With the extensive use of ICIs in cancer treatment of older patients, the question arises whether immunosenescence, the age-related decline in immune function, impacts the efficacy of ICI among older adults. Could these inhibitors hold a special significance for the aging population, perhaps by rejuvenating faltering immune responses, while concurrently navigating within a less resilient immune landscape that could potentially dampen their efficacy? The intersection of immunosenescence and immunotherapy reveals a multifaceted interplay warranting further comprehensive investigation. This commentary delves into our current understanding of the significance of immunosenescence markers and their potential utility as predictors for both treatment response and immune-related adverse events (irAEs) in older and frail cancer patients undergoing ICI therapy.

## 2 Immunosenescence in Older and Frail Adults

Frailty, characterized by increased vulnerability, stems from the progressive depletion of physiological reserves, influenced by both biological aging processes and the presence of age-related diseases^[4]^. A commonly employed definition of frailty in geriatric medicine describes it as a clinical condition marked by a decline in functioning across various physiological systems, accompanied by increased vulnerability to stressors which results in high risk of poor health outcomes, including falls, hospitalization and mortality^[5]^. A recent systematic review identified a consistent association between increased Interleukin-6 (IL-6) and C-reactive protein (CRP) serum levels and frailty^[6]^. Furthermore, a comprehensive cross-sectional study of peripheral blood mononuclear cells (PBMCs) in 1072 participants, revealed that elevated percentages of naïve CD4+ T cells and effector memory CD8+ T cells correlated with a reduced Frailty Index, whereas an increased percentage of CD8+ central memory T cells was linked to a higher Frailty Index^[7]^. These early studies suggest a correlation between the degree of frailty and immune competency.

A clearly defined or standardized set of parameters that can conclusively determine an individual’s status as ‘immunosenescent’ does not exist^[8]^. However, it has been established that the primary contributors to immunosenescence include senescent T cells and a distinctive inflammatory state marked by elevated pro-inflammatory markers, commonly referred to as ‘inflammaging’^[9]^.

Cancer and aging share a common origin in the progressive accumulation of cellular damage over time, driven by distinct yet interrelated mechanisms. The accrual of DNA damage triggers an increase in cell cycle inhibitors, inducing senescence or apoptosis. However, malignant cells bypass this regulatory process by acquiring further mutations, such as the deletion or inactivation of crucial tumour suppressors like p16INK4a or p53^[10]^. These changes cause cancer cells to have the capability to undergo unrestricted proliferation. Cellular senescence is characterized by irreversible cell-cycle arrest triggered by extensive replication. Senescent cells exhibit profound changes in chromatin and secretome, alongside increased expression of several senescence markers, such as Cdkn2a/p16Ink4a and Cdkn1a/p21Cip1^[11]^. Additionally, it is important to note that certain studies have indicated a dual role of senescent cells in tumorigenesis^[12]^. Some senescent cells restrain cancer cell progression, while others promote it. Moreover, both senescent and cancer cells upregulate anti-apoptotic factors, categorized as Senescent Cell Anti-Apoptotic Pathways (SCAPs), thereby enhancing their ability to evade apoptosis^[11]^.

The aging process impacts also the innate and adaptive branches of the immune system^[13]^. Aging is characterized by an expansion of terminally differentiated T cells while concomitantly experiencing a decline in the population of naïve CD8+ T cells^[14]^. Within older adults, naïve CD8+ T cells exhibit increased expression of markers associated with replicative senescence (CD8+CD45RA+CD27+), heightened susceptibility to apoptotic signals, and a constrained T cell receptor (TCR) repertoire^[15–17]^. One of the major age-associated changes that occur in the immune system is thymic involution, which leads to variations in the number of naïve T cells. This decline is particularly notable in CD8+ T cells compared to CD4+ T cells, as evidenced by the inversion of the CD4/CD8 ratio, a hallmark of immunosenescence^[18]^. Changes in this ratio, encompassing variations not only in CD8+ T-cells but also in CD4+ T-cells, could contribute to a state of mild activation, potentially compromising immune responses and exacerbating inflammation^[19]^.

Senescent T cells exhibit a Senescence-Associated Secretory Phenotype (SASP), characterized by the secretion of a spectrum of cytokines. This includes suppressive cytokines such as IL-10 and TGF-ß, alongside proinflammatory cytokines like TNF, IFNγ, IL-2, IL-6, and IL-8^[20,21]^. The SASP in T cells is regulated by p38 MAPK signaling, thereby contributing to age-related inflammation^[22]^. Furthermore, immunosenescence encompasses features such as dysregulated metabolism and epigenetic alterations^[9]^. With ageing, the immune system undergoes important changes due to lifetime exposure to external agents such as infections. For instance, a large proportion of CD8+ T cells in older adults are dedicated to managing chronic viral infections such as cytomegalovirus^[23]^. A significant area of research focuses on understanding the impact of aging on specific immune cell populations, particularly CD8+ T cells. Immunosenescence notably affects the PD-1/PD-L1 pathways, with CD8+ T cells being particularly influenced by this interaction^[23]^. T cell functional exhaustion is characterized by diminished functional capacity and increased expression of inhibitory receptors such as PD-1. This mechanism is crucial for regulating the extent of the effector T cell response but also contributes to the age-related decline in adaptive immune function^[24]^. As individuals age, PD-1 expression on T cells increases, and attempts to block this pathway do not fully restore T cell activity in older adults compared to younger individuals^[25–27]^. CD57 has been identified as the primary marker for replicative senescence, as evidenced by the considerable impairment in proliferative function observed in CD57-expressing T cells^[28]^. The replicative senescent phenotype is distinguished by several defining features, including shortened telomeres, absence of co-stimulatory molecules such as CD28 and CD27, and heightened expression of senescence-associated-β-galactosidase (SA-β-Gal)^[28]^. Another subset of senescent CD8+ T cells, characterized by the absence of both CD27 and CD28 expression alongside CD45RA expression, is termed effector memory T cells re-expressing CD45RA, commonly abbreviated as Temra cells^[29]^. The mechanism leading to their senescent state includes factors such as DNA damage resulting from reactive oxygen species (ROS)^[28]^. These senescent CD8+ T cells demonstrate compromised proliferative potential and, akin to CD4+ T cells, display diminished TCR diversity and reduced CD27 and CD28 expression, alongside the upregulation of CD57, killer cell lectin-like receptor subfamily G (KLRG1), Tim-3, Tight, and CTLA-4^[30–33]^.

Regulatory T cells (Tregs) also play a crucial role in shaping immune responses, particularly in the context of age-related changes. In older adults, Tregs show a notable increase in both quantity and immune suppressive efficacy compared to younger individuals^[34–37]^. Tregs are characterized by the expression of the CD4+ CD25+ markers, alongside the transcription factor Forkhead box P3 (Foxp3), which plays a pivotal role in their immunosuppressive function^[38,39]^. Furthermore, it’s important to acknowledge the presence of regulatory CD8+ T cells, albeit in smaller quantities. Similar to their CD4 counterparts, these cells exhibit immunosuppressive functions^[40]^. The inhibitory activity of CTLA-4 is partially mediated by enhancing the immunosuppressive activity of Tregs and by downregulating T helper cells. One such mechanism is through CTLA-4-dependent trogocytosis, wherein Tregs inhibit the T cell stimulatory function of antigen-presenting cells (APCs) by reducing their CD80/CD86 expression. This reduction in CD80/CD86 on APCs leads to dual suppressive effects on T cell immune responses: it limits CD80/CD86 costimulation specifically to naïve T cells and increases the availability of free PD-L1, which in turn suppresses PD-1-expressing effector T cells^[40]^.

## 3 Immunotherapy in Aging: Understanding Immunosenescence and its Impact on ICI Therapy Response and irAEs

The impact of immunotherapy is profound across a spectrum of immunogenic tumours, with a particularly striking effect observed in advanced melanoma cases, where approximately 50% of patients experience tumour regression and achieve long-term, durable control over cancer, a significant leap from the less than 10% historically achieved^[41]^. In current clinical use, the combination of PD-1/PD-L1 inhibitors with chemotherapy or targeted therapy is also frequently employed to treat various solid tumours, resulting in remarkable outcomes^[42]^. As checkpoint inhibitors aim to strengthen the T cell response to cancer treatments, this raises the questions how changes in T cell populations that are associated with immunosenescence can interfere with response to immunotherapy in frail older adults and if vital specific immunity is necessary for optimal efficacy. The efficacy of PD-1, PD-L1, and CTLA-4 inhibitors in older patients was explored through subgroup comparisons in randomized clinical trials, demonstrating an overall survival benefit comparable to that observed in young patients^[43,44]^. Moreover, consistent outcomes demonstrating similar treatment efficacy, have been observed in observational retrospective studies^[45,46]^. However, these studies did not explore the impact of immunosenescence on ICI efficacy and irAEs.

Numerous clinical trials have investigated a multitude of biomarkers to predict clinical outcomes in patients undergoing immunotherapy treatment^[47]^. However, currently, there is a lack of robust biomarkers. The increased expression of PD-L1 by tumour or infiltrating immune cells, high mutational loads and increased densities of tumour-infiltrating lymphocytes (TILs), are the most promising biomarkers that best correlate with efficacy of immunotherapy in some cancers^[47–50]^. Additionally, PD-L1 expression can be present on both tumour cells and immune cells. It serves as the most extensively validated, utilized, and recognized biomarker for directing patient selection to receive anti-PD-1 or anti-PD-L1 antibodies^[51]^. Despite its widespread use, PD-L1 remains an imperfect yet valuable predictive biomarker for response to anti-PD-1 or anti-PD-L1 antibodies across a range of tumour types^[51]^. Several articles discuss the presence or absence of markers, commonly associated not only with worse outcomes but also prevalent in immune senescence^[52]^. Hence, would it not be beneficial to delve into this area of research?

Clinically, previous studies showed that age in itself is not associated with a worse response to immunotherapy treatment^[53,54]^. However, these studies did not differentiate between patients with and without frailty, nor did they study immunosenescence as a predictive marker of response. Interestingly, there are several markers of immunosenescence that have been associated with response rates to treatment with immunotherapy. For example, in a small pilot study, the reduced expression of CD27 and CD28 markers, indicative of immune senescence, or the expression of senescent markers Tim-3 and CD57 on T cells in pretreatment peripheral blood mononuclear cells (PBMCs) of patients with metastatic melanoma, correlated with resistance to ICI^[55]^. Furthermore, in patients with non-small cell lung carcinoma, T cell immunosenescence observed in PBMCs-characterized by the loss of CD28 and the expression of CD57 and KLRG1 on peripheral CD8+ T cells-was associated with a worse efficacy of ICI^[56]^.

Besides the association between frailty and response, it is important to assess frailty in patients who are candidates for immunotherapy as several studies have showed a higher incidence of grade 1-2 irAEs in older patients, along with a trend towards early treatment discontinuation and a higher incidence of irAEs requiring treatment with immune-modulating medication^[57]^. These irAEs include gastrointestinal symptoms like colitis, endocrine dysregulation such as thyroiditis and adrenal insufficiency, and pulmonary complications like pneumonitis^[58]^. Notably, older patients exhibiting geriatric impairments appeared more susceptible to irAEs^[59]^. While clinical judgment remains irreplaceable and cannot be substituted by any geriatric scale, geriatric assessment is crucial for identifying eligible older patients for cancer therapy and providing valuable guidance for clinical treatment decisions and interventions in routine oncology practice^[60,61]^. Considering that the aging of the immune system does not strictly align with chronological age, it becomes imperative to evaluate if and how aging prompts heightened self-specific immunity in response to ICI. This assessment is essential for effectively identifying eligible patients for ICI therapy. The identification of such predictors for both irAEs and survival in frail patients is crucial, helping avoid unnecessary irAEs, given the potential impact on their overall quality of life^[62]^. For instance, dehydration may occur more rapidly with even mild colitis, causing a decline in renal function and subsequent hospitalization. This domino effect contributes to further sarcopenia due to reduced mobilization, escalating the susceptibility to falls and, consequently, the risk of fractures^[63]^. Preventing these unnecessary side effects by opting for monotherapy ICI treatment or even considering abstaining from treatment altogether will not only enhance the treatment experience or care for older and frail cancer patients but also contribute to a more cost-effective approach.

To our knowledge, no previous studies have investigated the interplay between frailty, immunosenescence, and response to ICI treatment. And thus, exploring this triad could provide valuable insights into optimizing therapeutic strategies and improving patient outcomes.

## 4 Immunosenescence in the TME

The TME, the focal point of the immune response against cancer, allows us to better understand the complex interplay between immunotherapy and anti-tumour immune responses. While analyzing peripheral blood offers practicality, disparities in immune cell markers between peripheral blood and the TME highlight the imperative for comprehensive evaluation^[51]^. Senescent cells become immunogenic by expressing stimulatory ligands such as MICA/B, which bind to NKG2D and trigger their destruction by NK cells and CD8+ T cell inhibition^[64]^. Additionally, senescent cells secrete chemokines and cytokines that attract immune cells into tissues, facilitating the clearance of these cells. However, this secretion process may also sustain a low-level chronic inflammatory state, contributing to many age-related diseases, such as cancer^[64]^. Despite the immune system’s ability to clear senescent cells, their accumulation during aging still occurs, likely due to a decline in immune function that results in the incomplete elimination of senescent cells with age^[64]^. The predominant characteristic of exhausted T cells within the TME is the heightened expression of a spectrum of inhibitory receptors, notably encompassing PD-1, CTLA-4, Tim-3, and LAG-3^[21]^. An increase in Treg within the TME is indicative of a poorer prognosis across many cancer types^[65–67]^. Multiple reports have assessed the correlation between intratumoural Tcell activation and regulation markers, investigating the association with TILs and response in cohorts treated with anti-CTLA-4, anti-PD-1, and combination immunotherapy^[68]^. These investigations have yielded significant findings^[68]^. In metastatic melanoma patients undergoing immunotherapy, high pretreatment levels of CD8 lymphocytic infiltration, determined both by in situ cell counts and protein expression via quantitative immunofluorescence, demonstrate significant associations with response, independent of various clinical factors^[69]^. The transcription factor TCF7, a component of the Wnt/β-catenin signaling pathway, plays a pivotal role in the formation of central memory, persistence, and self-renewal of CD8+ T cells^[70]^. Moreover, the TCF7 gene is hypermethylated and less expressed in CD8+ T cells from older individuals^[71]^. Its presence can predict positive clinical responses to ICI treatment in melanoma patients^[70]^.

Foxp3 is a master regulatory transcription factor for generating the immunosuppressive CD4+ Treg lineage. Treg infiltration in tumours contribute substantially to resistance against PD-1 blockade therapy in various cancer types^[72]^. As a key transcription factor, Foxp3 plays a central role in orchestrating the development of CD4+ Tregs, which exert these potent immunosuppressive effects^[72]^. A comprehensive knowledge of CD8+ T cell and CD4+ regulatory T cells senescence and the mechanisms underlying this process may be crucial for predicting response and adverse events in older patients treated with ICI.

## 5 Future Directions

Over the last decade, immunotherapies that modify both the immune components within the TME and in peripheral blood, effectively targeting and eliminating tumour cells, have revolutionized cancer treatment. However, despite the promising survival data, resistance to these treatments occurs in a large subset of patients. Furthermore, such treatments can induce severe, and occasionally life-threatening, irAEs. A limited number of studies have explored the correlation between immunosenescence markers in peripheral blood and the TME, frailty, and the response to ICI in older patient. Therefore, the impact of immunosenescence on both the response to ICI and irAEs remains uncertain, in particular due to the limited involvement of older cancer patients in clinical and translational studies exploring this facet. The focus on exploring the impact of immunosenescence on ICI efficacy and irAEs, as well as identifying reliable biomarkers, remains crucial for future research in geriatric oncology. Key questions for consideration include investigating whether immunosenescence contributes to lower efficacy, even in younger patients with an “older” immune system, and assessing the role of frailty as a cofactor in efficacy, including its potential impact on early therapy termination. Additionally, understanding the underlying causes of increased irAEs in frail patients and evaluating the balance between clinical benefit and quality of life loss due to these irAEs are essential. Addressing these aspects will aid in patient stratification and personalized treatment approaches, ultimately enhancing patient outcomes while minimizing the risks associated with irAEs.

## Data Availability

Not applicable.
